# The effect of aspartame and sucralose intake on body weight measures and blood metabolites: role of their form (solid and/or liquid) of ingestion

**DOI:** 10.1017/S0007114521003238

**Published:** 2022-07-28

**Authors:** Marie-Elizabeth E. Ragi, Rachelle El-Haber, Fidele El-Masri, Omar A. Obeid

**Affiliations:** Department of Nutrition and Food Sciences, Faculty of Agricultural and Food Sciences, American University of Beirut, Beirut 1107 2020, Lebanon

**Keywords:** Non-caloric sweeteners, Aspartame, Sucralose, Lean body mass, Body fat

## Abstract

The ingestion of non-caloric sweeteners (NCS) from food and/or drink was intended to reduce caloric intake without compromising palatability. However, the inconclusive relation between NCS and body weight may partially relate to their form of ingestion (solid or liquid). Thus, two paralleled experiments (aspartame and sucralose) were conducted. In each, Sprague Dawley rats (7-week-old male) were randomly divided into four groups. In Expt 1, aspartame (0·05 %) was added to the diet (AD) or drinking water (AW) or both diet and water (ADW), and a control group (C) was given a non-sweetened diet with plain water. In Expt 2, sucralose (0·016 %) was similarly provided in the diet (SD) or drinking water (SW) or both diet and water (SDW), with a control group (C). All rats had free access to food and water for 7 weeks. Energy intake, body weight and body composition were monitored and blood metabolites were determined. Results showed that aspartame ingestion significantly increased body weight and fat mass mainly due to an increase in energy efficiency. The effect was related to the amount rather than the form of ingestion. Additionally, aspartame ingestion was associated with glucose intolerance. Sucralose ingestion had a similar impact to that of aspartame though to a lesser extent. In conclusion, 7-week ingestion of aspartame and sucralose had adverse effects on body measures that were not related to the form of ingestion.

In the past decades, the increased consumption of sugar-sweetened beverages has been paralleled by an increase in weight gain, thus implicated in the development of obesity and its related diseases^(1–5)^. As a result, efforts directed towards the prevention of obesity, type 2 diabetes and CVD have been focused on lowering the consumption of sugar-sweetened beverages^(3,4)^. Surprisingly, the decline in the availability and intake of total added sugar was not associated with a decrease in the prevalence of obesity, which sustained its rise in the last couple of decades^(6,7)^.

The use of non-caloric sweeteners (NCS) has been established as a replacement strategy for sugar in sweetened beverages, notably in soft drinks. With an intense sweetening power and very low caloric contribution, they bring the sweet taste without the extra empty energy content and harmful effects attributed to sugars. In short-term studies, swapping sucrose-containing beverages with non-caloric beverages was shown to reduce weight gain and fat accumulation without resort to energy restriction^(8,9)^. However, some long-term studies showed a positive dose–response association between artificially sweetened beverages consumption with weight gain, incidence of type 2 diabetes and incidence of CVD^(10,11)^. These findings were proposed to relate to disturbances in the association between sweetness and energy intake. Normally, the sweet taste is thought to predict the caloric content of food. However, when sweetness is not accompanied by caloric intake, an adaptive mechanism is activated to compensate for caloric deficiency by increasing energy intake and/or diminishing energy expenditure^(12)^.

The relationship between sweetened (both caloric and non-caloric) beverages and body composition measures is far from being conclusive. The contrasting evidence mentioned above may suggest the involvement of other factors, such as ingestive behaviour. The form in which sweeteners are ingested, whether from solid food or liquid beverages, influences the metabolic processes. Indeed, energy-containing beverages were reported to elicit little dietary compensation as compared with solid food and may consequently lead to an increase in energy intake and a subsequent weight gain^(13)^. Therefore, we investigated, over a 7-week experimental period, the effect of NCS (aspartame and sucralose) ingestion from the diet (solid) and/or drinking beverages (liquid) on energy balance, body composition, and measures of metabolism in male Sprague Dawley rats.

## Methods

### Animal model

The experimental protocol (no. 18–02–453) was approved by the Institutional Animal Care and Use Committee (IACUC) of the American University of Beirut, Lebanon. The study was performed following the criteria outlined in the Guide for the Care and Use of Laboratory Animals. Male Sprague Dawley rats (7 weeks old, Animal Care Facility, American University of Beirut) were housed individually in wire-bottom cages in a temperature (22 ± 1°C) and light (reverse light cycle 12 h dark–12 h light, lights off at 10:00 h) controlled room. Food and liquids were offered *ad libitum* in containers and bottles easily accessible for the rats.

### Experimental design

Rats (*n* 48) were placed on a 1-week adaptation period, with free access to plain water and semi-synthetic AIN-93G-based diet (online Supplementary Table 1) to familiarise them with the environment and diet.

They were subsequently randomly divided into seven experimental groups, analysed as two separate experiments with the same control group. The amounts of sweeteners (0·05 % aspartame and 0·016 % sucralose) used were set to equal the sweetness level of 10 % sucrose solution, a common level of sweetness in beverages. The corresponding percentages – 0·05 % of aspartame and 0·016 % of sucralose – were added weight per weight in both diet and drink.

#### Expt 1

Effect of aspartame ingestion from water and/or diet on body weight measures and blood metabolites:

- Group C control (*n* 6): Rats were maintained on unsweetened starch-based diet and plain water
- Group AD (*n* 7): Rats were maintained on aspartame-sweetened diet and plain water
- Group AW (*n* 7): Rats were maintained on unsweetened starch-based diet, with aspartame-sweetened water offered for 12 h, followed by 12 h of plain water
- Group ADW (*n* 7): Rats were maintained on aspartame-sweetened diet, and aspartame-sweetened water offered for 12 h, followed by 12 h of plain water

#### Expt 2

Effect of sucralose ingestion from water and/or diet on body weight measures and blood metabolites:

- Group C control (*n* 6): Rats were maintained on unsweetened starch-based diet and plain water.
- Group SD (*n* 6): Rats were maintained on sucralose-sweetened diet with plain water
- Group SW (*n* 7): Rats were maintained on unsweetened starch-based diet, with sucralose-sweetened water offered for 12 h, followed by 12 h of plain water
- Group SDW (*n* 7): Rats were maintained on sucralose-sweetened diet, and sucralose-sweetened water offered for 12 h, followed by 12 h of plain water

Rats were fed *ad libitum* their respective diets and beverages (online Supplementary Table 1) for 7 weeks. In the groups where the rats were provided with a sweetened beverage (AW, ADW, SW and SDW), the respective sweetened water was only available for 12 h per d (dark phase), after which they were switched to regular drinking water to avoid potential osmolarity disturbances.

On the day of killing, overnight fasted rats were anesthetised with inhaled isoflurane (Forane®, Abbott,) administered at 4 % concentration (0·8 ml in a 4 l container). Blood was collected from the superior vena cava and rats were euthanised by severing their hearts. The livers, epididymal adipose tissue, kidneys and hearts were immediately excised, weighed, frozen in liquid N_2_ and stored at −80°C pending analysis. Blood samples were centrifuged at 2200 g (3°C) for 15 min, and aliquots of plasma were collected and stored at −80°C awaiting analysis.

### Food and fluid intakes, body weight and composition

Food and fluid intakes (difference in the weight of the food containers and water bottles) were measured twice per week. Body weight and body composition were assessed weekly using NMR minispec (LF110 BCA analyzer, Bruker). Body weight, lean body mass and body fat mass were expressed as gross weight. In order to minimise the impact of the variations in initial body weight within the groups, weight gain was presented by determining the changes from baseline. Energy stored was calculated as the energy retained in the body per 100 kJ of energy consumed (kJ/100 kJ), while energy expenditure was estimated from the total energy intake and changes in body mass and composition^(14)^.

Livers were freeze-dried (FreeZone 6 Freeze Dryer, LABCONCO) for 48 h, and hepatic fat content was determined by lipid extraction with petroleum ether (40–60°C) as solvent using an ANKOM XT10 extractor (ANKOM Technology). All determinations were carried out in duplicate.

### Plasma analysis

Fasting plasma glucose, total cholesterol, HDL-cholesterol, TAG, albumin, plasma urea nitrogen and creatinine were determined with Vitros 350 Chemistry System (Ortho-Clinical Diagnostics, Raritan). Plasma insulin concentration was measured by enzyme immunoassay using the Rat/Mouse Insulin ELISA Kit (EZRMI-13K) (EMD Millipore Corporation). The Homeostatic Model Assessment for Insulin Resistance (HOMA-IR) was calculated from the values of fasting plasma glucose and insulin^(15)^, by the formula: HOMA-IR = fasting plasma glucose (mmol/l) × fasting serum insulin (mU/l)/22·5.

### Statistical analysis

The required number of rats was calculated using previously determined weight gain data (6·0 ± 0·95 g/d) and assuming a 25 % difference in the mean, with a statistical power of 90 % and a 5 % significance level. Data were expressed as the mean and standard deviation of all values. SPSS Statistics 25.0 software (IBM Corp.) was used for statistical analysis. For both experiments, one-way ANOVA with sweetener type as a factor was performed. Multiple-way ANOVA (general linear model), using sweetener type and time as fixed factors, was used to analyse the results throughout the 7-week experimental period. In addition, Pearson’s correlation was performed to determine the relationship between the amounts of NCS ingested (aspartame or sucralose) with the different parameters assessed.

## Results

### Expt 1: Effect of aspartame ingestion from water and/or diet on body weight measures and blood metabolites

**Fig. 1.**
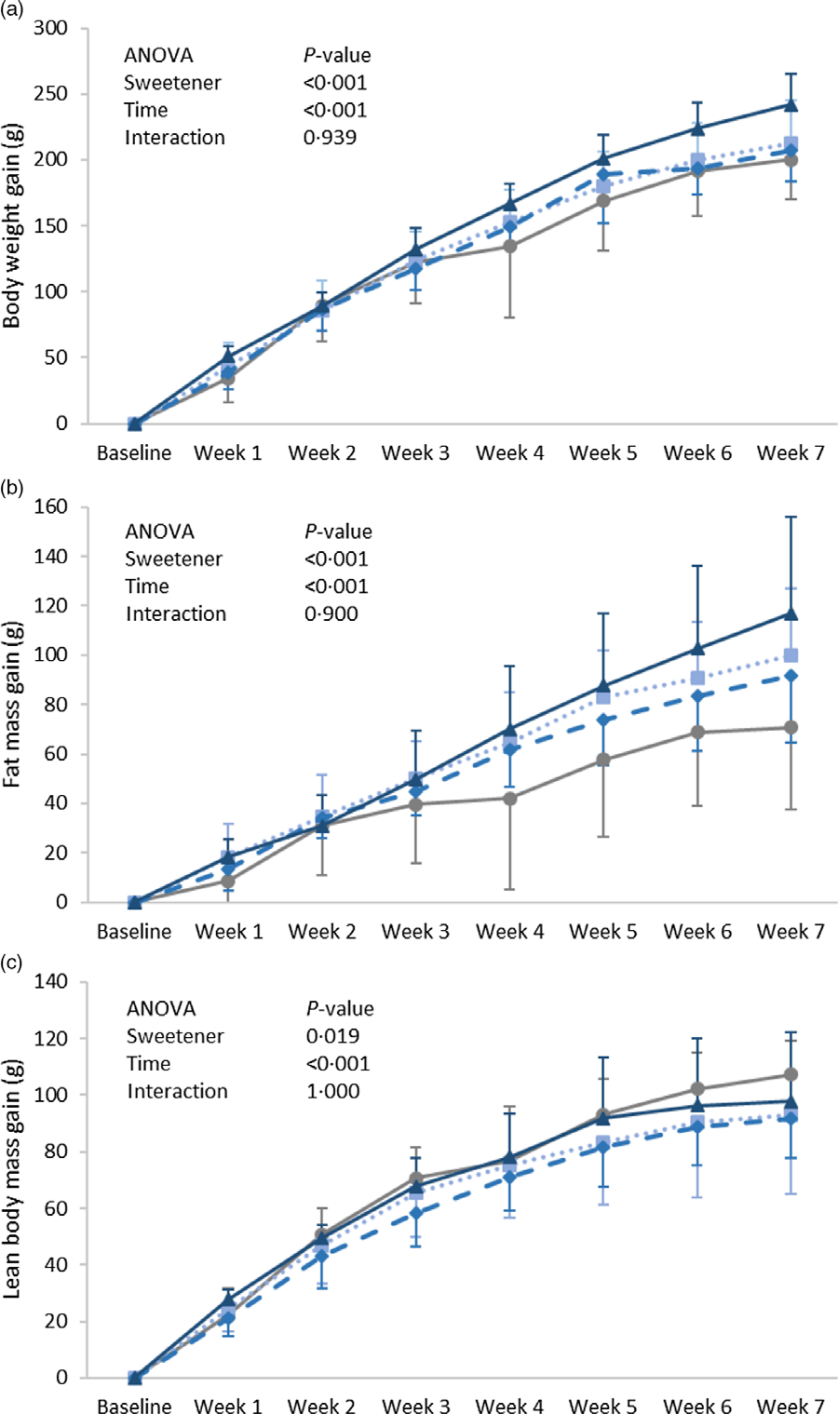
Expt 1 – weekly body weight (a), lean body mass (b) and body fat (c) of the four groups of rats in Expt 1 over the 8-week experimental period. Group C – control: starch-based diet and plain water; Group AD: aspartame-sweetened diet and plain water; Group AW: starch-based diet and aspartame-sweetened water; Group ADW: aspartame-sweetened diet and water. Data are expressed as the mean ± sd of all values. A two-way ANOVA was performed with time and group as factors. Significance was set at *P*-value < 0·05. 
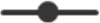
, C; 
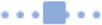
, AD; 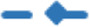, AW; 
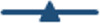
, ADW.

Body weight gain increased with time (*P* = 0·001) and showed a significant difference between groups (*P* = 0·001); it was found significantly higher in the ADW group as compared with the control group (Fig. 1(a)). A similar pattern was observed for fat mass gain, in which the significance between groups (*P* = 0·001) was highly attributed to a difference between the ADW and control groups (Fig. 1(b)). Lean body mass gain was significantly different according to time (*P* = 0·001) and groups (*P* = 0·019), with that of group ADW found lower than the control group but higher than that of group AW (Fig. 1(c)). In order to account for the amount of aspartame ingested per rat (Table 1) on body composition, a correlation was performed between ingested aspartame (whether from water and/or diet) and body weight measures. Interestingly, the amount of ingested aspartame was positively correlated with weight gain (*r* = 0·492, *P* = 0·009), fat mass gain (*r* = 0·430, *P* = 0·025) and energy efficiency (*r* = 0·395, *P* = 0·041).

**Table 1.** Expt 1 – effect of aspartame ingestion from water and/or diet on measures of energy balance

	C		AD		AW		ADW		*P*
	Mean	sd	Mean	sd	Mean	sd	Mean	sd	
Energy intake (kJ/d)	453·34	33·97	472·42	16·95	484·55	51·30	476·39	35·98	0·496
Fluid intake (g/d)	44·08	20·96	32·89	4·24	39·61	10·87	38·30	5·13	0·409
Plain water intake (g/d)	44·08	20·96^a^	32·89	4·24^a^	20·10	4·27^b^	19·58	2·20^b^	0·001
Sweet water intake (g/d)	0·00	0·00^a^	0·00	0·00^a^	19·51	7·19^b^	18·72	3·70^b^	0·001
Sweetener intake (mg/d)	0·00	0·00^a^	14·86	0·53^b^	9·76	3·60^c^	24·34	2·08^d^	0·001
Energy expenditure (kJ/d)	387·48	38·74	384·13	22·51	403·09	42·05	374·18	50·75	0·601
Energy stored (kJ/100kJ)	14·51	5·57	17·94	4·00	16·73	3·58	21·62	6·64	0·107

Group C – control: starch-based diet and plain water; Group AD: aspartame-sweetened diet and plain water; Group AW: starch-based diet and aspartame-sweetened water; Group ADW: aspartame-sweetened diet and water.

Data are expressed as the mean ± sd of all values. A one-way ANOVA was performed, data with the same subscript are not significantly different according to Fisher’s pairwise comparison.

Significance was set at a *P*-value < 0·05.

At the energy balance level, energy intake (*P* = 0·496), expenditure (*P* = 0·601) and efficiency (*P* = 0·107) were similar between the groups (Table 1). However, energy efficiency of the ADW was slightly higher and a significant positive correlation was found with the amount of ingested aspartame (*r* = 0·410, *P* = 0·034). Total fluid intake (*P* = 0·409) was not affected by aspartame ingestion. The amount of aspartame consumed was, however, significantly different, highest when the sweetener was added to both diet and drink (group ADW), followed by group AD with aspartame in the diet, then lowest in group AW when aspartame was given in the water alone (*P* < 0·001).

Liver wet weight differed between groups (*P* = 0·022), with group ADW showing the highest weight, while the percentages of water and fat in the liver were similar between the groups (Table 2). Moreover, epididymal adipose tissue weight of the group ADW was significantly higher than that of the other groups (*P* = 0·007) and positively correlated with the amount of ingested aspartame (*r* = 0·559, *P* = 0·002). The weights of the kidneys and hearts of all rats were found to be similar, with no significant differences observed between the four groups (online Supplementary Table 2).

**Table 2.** Expt 1 – effect of aspartame ingestion from water and/or diet on weight of organs and plasma metabolites

	C		AD		AW		ADW		*P*
	Mean	sd	Mean	sd	Mean	sd	Mean	sd	
Liver wet weight (g)	12·48	0·69^a^	14·37	2·15^ab^	12·61	1·91^a^	15·53	2·38^b^	0·022
Liver weight % of BW (g/100g BW)	2·54	0·13	2·73	0·26	2·50	0·24	2·78	0·25	0·085
Liver water (%)	67·52	2·15	67·55	2·36	66·06	3·87	67·71	1·01	0·514
Liver fat in wet (%)	5·84	1·86	5·48	1·36	6·31	1·16	7·21	0·86	0·118
Epididymal AT weight (g)	8·78	2·46^a^	11·52	2·12^a^	11·08	2·06^a^	14·55	3·62^b^	0·007
Fasting plasma glucose (mg/dl)	166·67	40·76^a^	244·14	48·42^bc^	218·00	47·30^ab^	268·14	41·50^c^	0·003
Fasting plasma insulin (ng/ml)	0·55	0·74	1·02	0·65	0·58	0·72	1·39	0·73	0·125
HOMA-IR	7·41	11·10^b^	17·73	10·26^ab^	9·72	13·50^b^	25·61	12·23^a^	0·040
Fasting plasma TAG (mg/dl)	46·17	21·68^a^	65·14	28·79^ab^	43·14	8·43^a^	74·57	22·87^b^	0·039

BW, body weight; AT, adipose tissue; HOMA-IR, Homeostatic Model Assessment for Insulin Resistance.

Group C – control: starch-based diet and plain water; Group AD: aspartame-sweetened diet and plain water; Group AW: starch-based diet and aspartame-sweetened water; Group ADW: aspartame-sweetened diet and water.

Data are expressed as the mean ± sd of all values. A one-way ANOVA was performed, data with the same subscript are not significantly different according to Fisher’s pairwise comparison.

Significance was set at a *P*-value < 0·05.

At the blood metabolites level, fasting plasma glucose was significantly higher among the aspartame groups (*P* = 0·003) and this seems to relate to the amount of ingested aspartame (*r* = 0·585, *P* = 0·001) (Table 2). Fasting plasma insulin levels failed to reach significance between the groups (*P* = 0·125), whereas HOMA-IR was significantly different between groups (*P* = 0·04) and strongly related to the amount of ingested aspartame (*r* = 0·518, *P* = 0·006), and thus insulin sensitivity was inversely related to aspartame intake. Fasting plasma TAG levels were also significantly different between the groups (*P* = 0·039), in which AD and ADW had the highest levels. As for the remaining parameters, no significant differences were detected between all four groups for total cholesterol, HDL, albumin, plasma urea nitrogen and creatinine levels (online Supplementary Table 2).

### Expt 2: Effect of sucralose ingestion from water and/or diet on body weight measures and blood metabolites

**Fig. 2.**
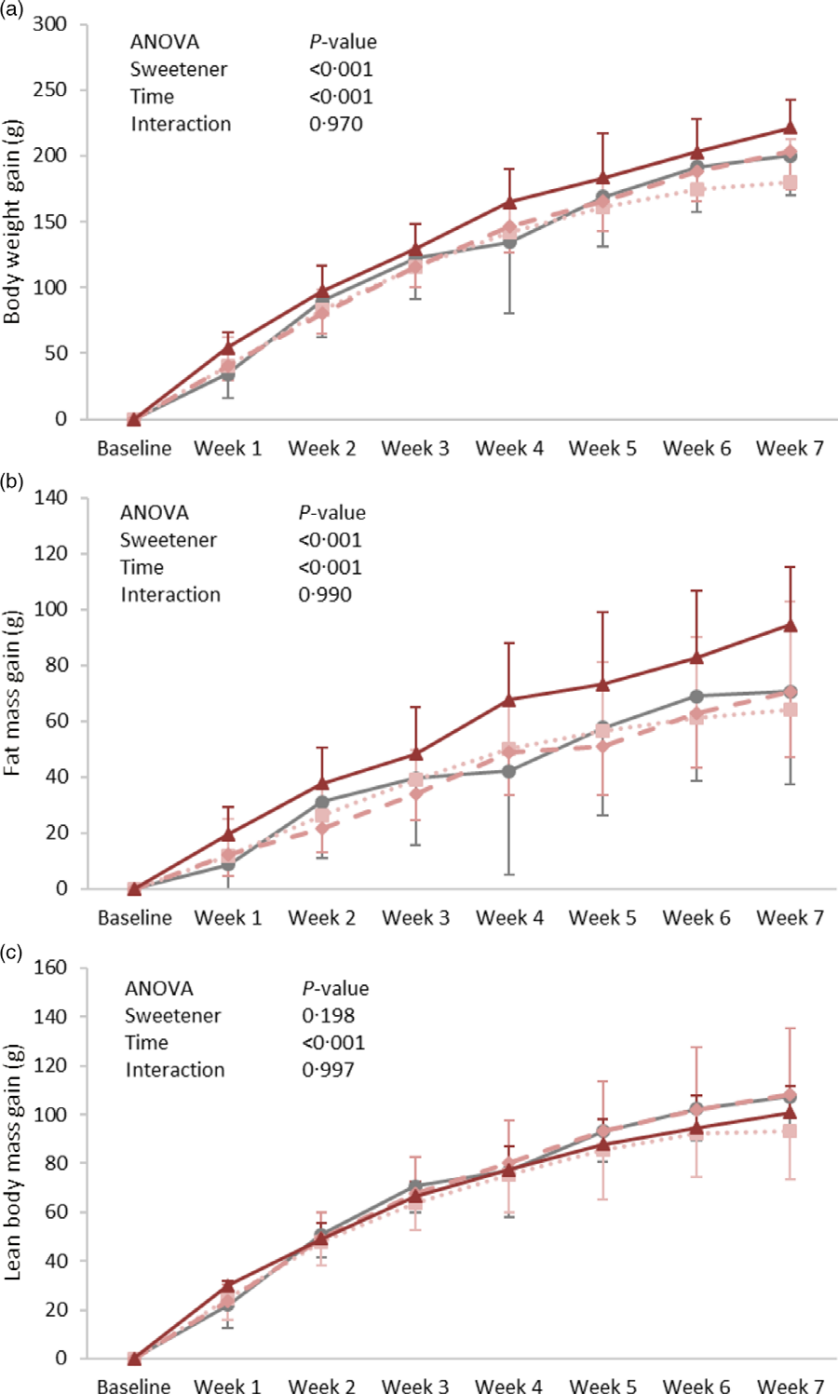
Expt 2 – weekly body weight (a), lean body mass (b) and body fat (c) of the four groups of rats in Expt 1 over the 8-week experimental period. Group C – control: starch-based diet and plain water; Group SD: sucralose-sweetened diet and plain water; Group SW: starch-based diet and sucralose-sweetened water; Group SDW: sucralose-sweetened diet and water. Data are expressed as the mean and standard deviation of all values. A two-way ANOVA was performed with time and group as factors. Significance was set at *P*-value < 0·05. 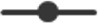, C; 
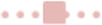
, SD; 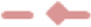, SW; 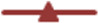, SDW.

Body weight gain showed statistical significance according to time (*P* = 0·001) and groups (*P* < 0·001), mainly due to a higher weight gain in group SDW as compared with SD (Fig. 2(a)). Similarly, fat mass gain was significant according to time (*P* = 0·001) and groups (*P* < 0·001). Fat gain of the SDW group was significantly higher than that of the SW group (Fig. 2(b)), whereas lean body mass gain was found similar between the varied groups (*P* = 0·474) (Fig. 2(c)). However, no significant correlation was detected between the amount of ingested sucralose and body weight gain, fat mass gain, or lean body mass gain.

Moreover, all measures of energy balance were similar between the groups, with energy intake, expenditure and efficiency failed to reach statistical significance (Table 3). Also, no difference in total fluid intake was observed between the groups (Table 3). Liver weight and composition, as well as epididymal fat weight, were all similar between the groups (Table 4). No statistical significance was detected in any of the blood parameters (Table 4 and online Supplementary Table 3). However, fasting plasma glucose of the sucralose groups was slightly higher than that of the control (Table 4), while insulin and HOMA-IR were similar between the groups. Total and HDL-cholesterol both showed a pattern whereby higher levels were found in the SD group as compared with either the SW or SDW groups, but with no statistical significance detected (online Supplementary Table 3).

**Table 3.** Expt 2 – effect of sucralose ingestion from water and/or diet on measures of energy balance

	C		SD		SW		SDW		*P*
	Mean	sd	Mean	sd	Mean	sd	Mean	sd	
Energy intake (kJ/d)	453·3	34·0	439·9	27·5	437·2	32·1	474·0	25·2	0·118
Fluid intake (g/d)	44·08	20·96	36·57	6·85	34·10	9·71	38·23	12·38	0·599
Plain water intake (g/d)	44·08	20·96^a^	36·57	6·85^a^	18·30	4·37^b^	19·88	5·82^b^	0·001
Sweet water intake (g/d)	0·00	0·00^a^	0·00	0·00^a^	15·79	6·83^b^	18·35	8·11^b^	0·001
Sweetener intake (mg/d)	0·00	0·00^a^	4·43	0·0·23^b^	2·53	1·09^c^	7·71	1·10^d^	0·001
Energy expenditure (kJ/d)	387·5	38·7	380·6	46·4	371·3	34·2	389·6	17·0	0·769
Energy stored (kJ/100kJ)	14·51	5·57	13·62	7·04	15·09	4·05	17·73	2·91	0·493

Group C – control: starch-based diet and plain water; Group SD: sucralose-sweetened diet and plain water; Group SW: starch-based diet and sucralose-sweetened water; Group SDW: sucralose-sweetened diet and water.

Data are expressed as the mean ± sd of all values. A one-way ANOVA was performed, data with the same subscript are not significantly different according to Fisher’s pairwise comparison.

Significance was set at a *P*-value < 0·05.

**Table 4.** Expt 2 – effect of sucralose ingestion from water and/or diet on weight of organs and plasma metabolites

	C		SD		SW		SDW		*P*
	Mean	sd	Mean	sd	Mean	sd	Mean	sd	
Liver wet weight (g)	12·48	0·69	13·49	2·27	13·11	2·19	15·19	3·42	0·227
Liver weight % of BW (g/100g BW)	2·54	0·13	2·73	0·30	2·65	0·28	2·82	0·45	0·440
Liver water (%)	67·52	2·15	67·61	2·78	70·85	5·34	71·26	6·29	0·320
Liver fat in wet (%)	5·84	1·86	6·18	2·49	5·88	3·57	5·71	1·76	0·990
Epididymal AT weight (g)	8·78	2·46	10·00	3·52	8·87	2·10	12·08	3·02	0·137
Fasting plasma glucose (mg/dl)	166·7	40·8	204·0	32·0	207·6	39·3	258·0	93·3	0·068
Fasting plasma insulin (ng/mL)	0·55	0·74	1·03	0·88	0·67	1·09	0·99	1·01	0·758
HOMA-IR	7·41	11·10	17·23	14·96	11·32	20·38	17·56	16·85	0·646
Fasting plasma TAG (mg/dl)	46·17	21·68	40·00	17·11	35·29	9·03	51·57	20·78	0·367

BW, body weight; AT, adipose tissue, HOMA-IR, Homeostatic Model Assessment for Insulin Resistance.

Group C – control: starch-based diet and plain water; Group SD: sucralose-sweetened diet and plain water; Group SW: starch-based diet and sucralose-sweetened water; Group SDW: sucralose-sweetened diet and water.

Data are expressed as the mean ± sd of all values. A one-way ANOVA was performed, data with the same subscript are not significantly different according to Fisher’s pairwise comparison.

Significance was set at a *P*-value < 0·05.

## Discussion

NCS have been introduced to diets with the intention of reducing caloric intake and normalising blood glucose levels without compromising diet palatability. They have been extensively used as a substitute for sugar, providing the sweet taste without any energy load, and resulting in short-term reductions in body weight and fat^(8,9)^. However, their use coincides with the increase in the obesity and diabetes epidemics, and long-term studies associated them with weight gain and designated them as a factor fuelling obesity^(11,12,16–19)^. Indeed, prolonged consumption of foods or fluids containing acesulfame-K or saccharin, in comparison with glucose, has led to an increase in food intake, body weight and body fat accumulation in rats^(20,21)^. Our previous work further supported this notion, as both body weight and fat gains were found to be higher in rats consuming acesulfame-K as compared with sucrose-sweetened water^(22)^. Meanwhile, the ingestion of dietary carbohydrates in varied forms (solid food or liquid beverages) was reported to influence metabolic processing and body composition differently^(13)^. Accordingly, we designed an experiment to investigate the impact of the ingestion of NCS (aspartame and sucralose) in varied forms on body weight measures. In the current study, the amount of sweeteners used were set to equal the sweetness level of a 10 % glucose solution, a common level used in beverages.

Aspartame ingestion for 7 weeks was able to increase body weight gain, in which gain was positively associated with the amount of ingested aspartame rather than the form of aspartame (water or diet) ingestion. The increase in weight gain was highly attributed to total body fat gain. This was also apparent in the varied organs, where group ADW had high liver fat (%) and epididymal fat pad weight. The similarity in energy intake and energy expenditure between the groups implies that such an increase was related to improved energy efficiency, which was also found to be positively associated with the amount of ingested aspartame. These observations are in line with other studies in which weight gain following long-term consumption of aspartame-sweetened yogurt was not associated with an increase in caloric intake^(16,23)^. Thus, it can be concluded that weight gain was highly attributed to an enhancement in energy efficiency.

On the other hand, the impact of sucralose ingestion for 7 weeks was similar to that of aspartame. Body weight measures (weight gain, fat mass gain and lean body mass gain) were increased, though to a lesser magnitude. This may have been behind the failure to show any significant correlation between these parameters and sucralose ingestion. Liver and epididymal fat weight measures also failed to reach any significant difference between groups. Similar to aspartame, sucralose ingestion was not found to affect energy intake and expenditure.

Looking at the figures from both experiments, it is worth noting that the changes in weight and fat gain were manifested from week 3 and this implies that the impact of aspartame and sucralose is not acute and may be related to an up-regulation of parameters involved in lipogenesis and/or fat storage. In fact, the consumption of aspartame-sweetened beverages was linked to extensive liver damage and possible stimulation of hepatic lipogenesis leading to fat deposition in adipose tissues^(24)^. Our findings are in accordance with the numerous studies associating the long-term consumption of NCS with weight gain, further confirming the poor impact of NCS on body composition and metabolism^(11,12,18–21)^. Indeed, NCS were proposed to disrupt the caloric signal associated with sweetness, and thus its failure to provide energy content may consequently enhance energy deposition through the triggering of the starvation mode, which is known to reduce energy expenditure and increase in energy efficiency^(12)^. NCS are actually recognised by the sweet taste receptors that are expressed throughout the body; they however do not seem to be associated with potent satiety signals^(25)^. With animals using sweet taste to predict the caloric contents of food, eating sweet non-caloric substances – that brings the sweet taste without the energy content – will create a positive energy balance through increased food intake and/or diminished energy expenditure^(12)^. Moreover, aspartame and sucralose were reported to have different sites of action on the sweet taste receptors. Indeed, sucralose, like acesulfame K and sucrose, bind to both T1R2 and T1R3 sites of the sweet taste receptors, while aspartame only binds to T1R2^(25)^. The ability of both aspartame and sucralose to affect body weight and fat raises questions about the involvement of sweet taste receptors in the process of weight gain. In support, the artificial sweeteners saccharin and acesulfame-K were reported to stimulate adipogenesis and suppress lipolysis in a mechanism that is independent of sweet taste receptors^(26)^. Furthermore, the inability of rodents to detect the sweet taste of aspartame, in contrast to that sucralose^(25)^, as well as the failure of both aspartame and sucralose to increase caloric intake or decrease energy expenditure, do not support the concept of sweet-caloric detection^(12)^. It is worth noting that in both experiments the ingestion of aspartame or sucralose (whether from water and/or diet) was not found to affect the proportion of fluid intake from plain to sweetened water, in contrast to that reported previously, in which caloric (sucrose) and non-caloric (acesulfame K)-sweetened water intake was highly favoured over plain water^(22)^. In the current study, the mechanism by which NCS affect body weight seems to relate to an improvement in energy efficiency, though the mechanism behind such improvement remains to be elucidated.

Insulin sensitivity (*r* = 0·518, *P* = 0·006) was negatively and both fasting plasma glucose (*r* = 0·585, *P* = 0·001) and insulin (*r* = 0·442, *P* = 0·021) were positively correlated with aspartame ingestion. These changes may have contributed to the increase in plasma TAG (*r* = 0·437 *P* = 0·023) and epididymal fat pad weight (*r* = 0·559, P = 0·002). However, the modest increase in plasma glucose of the sucralose groups did not reach statistical significance. As previously mentioned, the consumption of NCS was intended as a substitute to sugar that does not affect glycaemia^(25)^, but recent data reported conflicting evidence and associated NCS consumption with increased blood glucose levels and risk of type 2 diabetes^(17,27)^. While the mechanisms responsible for the impact of NCS on body metabolism remain unclear, many theories have been laid to explain the adverse effect on glucose homoeostasis. On the one hand, aspartame consumption in water by mice was reported to inhibit endogenous intestinal alkaline phosphatase activity, which is known to prevent the metabolic syndrome, thus leading to increment in weight gain and plasma glucose, impaired glucose tolerance, and increased TNF-*α* levels^(28)^. Additionally, recent data are focusing on the possible involvement of circulating metabolites and alternations to the gut microbiota as driving factors behind the effect of NCS on glucose metabolism^(28,29)^. Chronic high doses of aspartame in rats induced an increase in fasting glycaemia and an impairment in insulin-stimulated glucose disposal. These were paralleled with alterations in the gut microbiota and an increase in SCFA propionate, a highly gluconeogenic substrate associated with inflammation and insulin resistance^(29)^. The observed increase in liver fat content in the ADW group may also partially contribute to aspartames’ reported suspicion of inflicting damages on many organs, notably the liver, through its role in provoking oxidative stress and increasing free radical production and pro-inflammatory cytokines^(30)^.

In conclusion, 7-week aspartame ingestion was found to increase body weight gain mainly due to fat accumulation, more specifically from week three and onwards. These effects were not accompanied by changes in energy intake or expenditure and thus relate to an enhancement in energy efficacy. Additionally, insulin sensitivity was reduced due to an increase in both glucose and insulin levels, while liver and epididymal fat weights were increased. These changes were dose-dependent, and thus with the differences observed in the intake of sweeteners (both aspartame and sucralose) between groups, the impact of the form of ingestion (diet and/or water) remains unclear. Sucralose was found to have a similar effect to that of aspartame, though to a lower extent. Our findings highlight the adverse effects of NCS aspartame and sucralose on body composition and metabolism.

## Supplementary material

For supplementary material accompanying this paper visit https://doi.org/10.1017/S0007114521003238. click here to view supplementary material
