# Supplementary material for: The effect of aspartame and sucralose intake on body weight measures and blood metabolites: role of their form (solid and/or liquid) of ingestion
Source: Br J Nutr. 2021 Aug 23;128(2):352–60. doi: 10.1017/S0007114521003238 (PMC9301525; doi:10.1017/S0007114521003238)
Supplement: Supplementary file 1 [file S0007114521003238sup001.docx]

**Supplementary Table 1.** Composition of diets and beverages provided to rats for 8 weeks. Composition of starch-based, aspartame-sweetened, and sucralose-sweetened diets as well as aspartame and sucralose-sweetened water.

| **Ingredients** | **Quantity (g/Kg)** | | | | | | |
| --- | --- | --- | --- | --- | --- | --- | --- |
|  | **Adaptation Diet** | | **Starch-Based Diet** | | **Aspartame-Sweetened Diet (0.05%)** | | **Sucralose-Sweetened Diet (0.016%)** |
| Casein | 200 | | 200 | | 200 | | 200 |
| L-methionine | 3 | | 3 | | 3 | | 3 |
| Starch | 532 | | 632 | | 632 | | 632 |
| Sucrose | 100 | | 0 | | 0 | | 0 |
| Aspartame | 0 | | 0 | | 0.5 | | 0 |
| Sucralose | 0 | | 0 | | 0 | | 0.16 |
| Oil | 70 | | 70 | | 70 | | 70 |
| Cellulose | 50 | | 50 | | 50 | | 50 |
| Mineral Mix | 35 | | 35 | | 35 | | 35 |
| Vitamin Mix | 10 | | 10 | | 10 | | 10 |
| Total Weight | 1000 | | 1000 | | 1000 | | 1000 |
|  | **-** | **Regular Water** | | **Aspartame-Sweetened Water (0.05%)** | | **Sucralose-Sweetened Water (0.016%)** | |
| Aspartame | **-** | 0 | | 0.5 | | 0 | |
| Sucralose | **-** | 0 | | 0 | | 0.16 | |

**Supplementary Table 2.** Experiment 1 – Effect of aspartame ingestion from water and/or diet on weight of organs and plasma metabolites

|  | C | AD | AW | ADW | *P*-value |
| --- | --- | --- | --- | --- | --- |
| Initial body weight (g) | 320.5±24.3 | 320.7±17.2 | 323.2±24.3 | 321.6±26.4 | 0.996 |
| Heart weight (g) | 1.42±0.14 | 1.54±0.19 | 1.46±0.08 | 1.50±0.11 | 0.406 |
| Kidneys weight (g) | 3.16±0.31 | 3.09±0.24 | 3.12±0.28 | 3.19±0.30 | 0.928 |
| Total-cholesterol (mg/dl) | 64.83±10.17 | 68.43±10.61 | 66.71±9.89 | 74.43±9.93 | 0.360 |
| HDL-cholesterol (mg/dl) | 42.83±8.42 | 47.43±8.87 | 45.86±6.62 | 49.57±5.83 | 0.446 |
| Albumin (g/dl) | 3.38±0.17 | 3.31±0.21 | 3.47±0.21 | 3.46±0.28 | 0.549 |
| Plasma urea nitrogen (mg/dl) | 13.67±1.37 | 13.71±2.29 | 14.29±2.36 | 13.57±1.99 | 0.916 |
| Creatinine (mg/dl) | 0.25±0.08 | 0.24±0.05 | 0.26±0.08 | 0.23±0.05 | 0.875 |

Group C – Control: starch-based diet and plain water; Group AD: aspartame-sweetened diet and plain water; Group AW: starch-based diet and aspartame-sweetened water; Group ADW: aspartame-sweetened diet and water

Data are expressed as the mean ± SD of all values. A one-way ANOVA was performed, data with the same subscript are not significantly different according to Fisher’s pairwise comparison.

Significance was set at a *P-value*<0.05.

**Supplementary Table 3.** Experiment 2 – Effect of sucralose ingestion from water and/or diet on weight of organs and plasma metabolites.

|  | C | | SD | | SW | | SDW | | *P*-value |
| --- | --- | --- | --- | --- | --- | --- | --- | --- | --- |
| Initial body weight (g) | | 320.5±24.3 | | 320.2±24.4 | | 321.9±26.7 | | 320.2±26.9 | 0.999 |
| Heart weight (g) | 1.42±0.14 | | 1.41±0.11 | | 1.47±0.14 | | 1.44±0.18 | | 0.899 |
| Kidneys weight (g) | 3.16±0.31 | | 3.04±0.34 | | 3.17±0.41 | | 3.19±0.42 | | 0.890 |
| Total-cholesterol (mg/dl) | 64.83±10.17 | | 70.00±5.48 | | 61.86±6.94 | | 59.71±10.95 | | 0.204 |
| HDL-cholesterol (mg/dl) | 42.83±8.42 | | 49.83±4.54 | | 41.43±4.65 | | 41.29±10.42 | | 0.173 |
| Albumin (g/dl) | 3.38±0.17 | | 3.25±0.36 | | 3.47±0.38 | | 3.37±0.26 | | 0.641 |
| Plasma urea nitrogen (mg/dl) | 13.67±1.37 | | 13.83±1.17 | | 13.43±3.41 | | 14.43±1.51 | | 0.840 |
| Creatinine (mg/dl) | 0.25±0.08 | | 0.25±0.08 | | 0.26±0.08 | | 0.23±0.05 | | 0.900 |

Group C – Control: starch-based diet and plain water; Group SD: sucralose-sweetened diet and plain water; Group SW: starch-based diet and sucralose-sweetened water; Group SDW: sucralose-sweetened diet and water

Data are expressed as the mean ± SD of all values. A one-way ANOVA was performed, data with the same subscript are not significantly different according to Fisher’s pairwise comparison.

Significance was set at a *P-value*<0.05.
